# On a New Mode of Treating Deafness, Attended by the Loss of the Membrana Tympani, Associated, or Not, with Discharge from the Ear

**Published:** 1848-09

**Authors:** James Yearsley

**Affiliations:** Surgeon to the Metropolitan Ear Institution


*On a new mode of treating Deafness, attended by loss of the Membrana Tympani, associated, or not, with discharge from the Ear.* By James Yearsley, Esq.. M. R. C. S., Surgeon to the Metropolitan Ear Institution.—Up to the present time, no successful mode of treating perforations of the membrane of the drum of the ear, either as respects the restoration of the membrane, or the relief of the accompanying deafness, has been discovered. The only means resorted to for the latter purpose have been the removal of pus or mucus from the tympanal cavity, by syringing or rendering it free by passing air through the perforation, by way of the Eustachian tube. Either of these proceedings will often produce a temporary improvement of the hearing in cases where the tympanum suffers from obstruction, but in many others, when such a state does not obtain, they are of little, if of any service. Another mode of treatment, but one directed to the renovation of the drum, is the employment of mercury, with a view to produce "a new creation of membrane." The translator of Kramer's work (Dr. R. Bennett) has referred to such a case, but it was not attended by results that would justify others in pursuing a similar treatment. In this case, one-fourth of the membrane was gone, but, under the influence of mercury (?) a thin pellicle extended over the aperture, which led to the hope that an artificial membrane would form. But, in the first place, I believe a patient would be in much better condition, with a loss of one-fourth of the membrane, than with an artificial membrane or a cicatrix ; in the next, I doubt whether the supposed membrane was anything more than a film of mucus, such as frequently fills up a perforation in the drum ; at all events, it disappeared as mysteriously as it had formed. The hearing was at first stimulated, but, after the mercurial course, the auditory organ gradually became, as is usual in such cases, more and more torpid.

I have now, however, the extreme gratification of promulgating a mode of relief for deafness attended by loss of the membrana tympani, which will cause great surprise among the readers of the Lancet, not less from its extreme simplicity, than from the extraordinary success which generally attends its employment.

In 1841, a gentleman came from New York, to consult me under the following circumstances :—He had been deaf from an early age, and on examination, I found great disorganization of the drum of each year. On my remarking this to him, he replied, "How is it, then, that, by the most simple means, I can produce on the left side a degree of hearing quite sufficient for all ordinary purposes ; in fact, so satisfied am I with the improved hearing which I can myself produce, that I only desire your assistance on behalf of the other ear." Struck by his remark, I again made a careful examination of each ear, and observing their respective conditions, I begged him to show me what he did to that ear, which I should unhesitatingly have pronounced beyond the reach of remedial art. I was at once initiated into the mystery, which consisted of the insertion of a spill of paper previously moistened at its extremity with saliva, which he introduced to the bottom of the meatus, the effect of which, he said, was "to open the ear to a great increase of hearing." This improvement would sometimes continue an hour, a day, or even a week, without requiring a repetition of the manipulation. Such an interesting fact could not fail to excite my attention, and it naturally occurred to me to try so simple a method in other cases. I did so in several which appeared to me to be identical with that of my patient, but I invariably failed. I was on the point of abandoning the idea that the remedy could ever be made available in practice, and of considering either that my American patient's case was unlike all others, or that it depended on some idiosyncrasy, when it happened that a young lady came under my care, by the recommendation of Mr. Squib, surgeon, of Orchard street. She was the daughter of wealthy parents, whose anxiety for her relief was so great as to induce them to bring her to me long after I had discouraged their visits, and openly expressed my inability to relieve her. She had become deaf at a very early age, after scarlatina, which had produced disorganization of the drum of each ear, and the deafness was extreme. Unwilling, however, to abandon hope, her friends continued to bring her to me, in order, as they said, that "nothing might be left untried." With little expectation of success, after so many previous failures, I was induced to apply the new remedy, with some modifications upon my previous experiments. Instead of adopting my American patient's plan, it occurred to me to try the effect of a small pellet of moistened cotton wool, gently inserted and applied at the bottom of the meatus, so as to come in contact with the small portion of membrane which still remained. The result was astoundingly successful. On the evening of a day in which she had risen from her bed with the sad reflection that she must be for ever debarred from social converse and enjoyment, she joined the family dinner-party, and heard the conversation which was going on around her with a facility that appeared to all present quite miraculous. Day after day, the remedy was applied with the same marked success, and eventually she learned the art of applying it herself, and thus became independent of me. It was observed that, until the wool could be brought in contact with a particular spot at the bottom of the meatus, the hearing was not at all benefited; on the contrary, was prejudiced: but the moment it was properly adjusted on that particular spot, the hearing was restored. Subsequent experience, in a vast number of cases, confirms this remarkable fact. It is not merely necessary to insert moistened cotton wool to the bottom of the meatus. Such a manipulation would in most cases add to the deafness. It is essential to find the spot on which to place the wool, and so adjust it as to produce the best degree of hearing of which the case may happen to be susceptible. This of course differs according to the variety and extent of the disorganization.

I quote the above case, not only because it was the first which it was my happiness to relieve by this novel plan, but because I am in a position to show the permanency of the remedy; for recently I have made it my business to write to the mother of the young lady, who states that her daughter "continues to derive the same benefit as ever from the remedy, and that in her case it has been most successful, restoring her to the charms of society, from which she had been almost entirely excluded. It is now scarcely necessary for the members of her family to raise their voices when addressing her." She adds: "When the aid is removed she scarcely hears at all."

For nearly five years this young lady has used the remedy with undiminished success, and during the same period I have been availing myself of it in the ordinary routine of my practice, stepping neither to the right nor to the left to seek for cases in which it would be applicable, nor ever speaking of its extraordinary success out of the circle of my immediate medical acquaintance. And most probably I should have continued so to do, if it had not happened that a gentleman, an army surgeon, recently consulted me, who having experienced the most happy result from the same mode of treatment, thought proper to publish some account of it in a local newspaper, considering, as he stated, that so important a mode of treatment ought to be more extensively known.

Mr. Griffiths, of Pantgwyn, Newcastle Emlyn, Carmarthenshire, the gentleman in question, (I am at liberty to use his name,) did me the honour to call on me in September of last year, accompanied by Sir David Davies, to consult me about a young friend labouring under an affection of the throat. During the consultation it was necessary for me to raise my voice very considerably to make myself heard by Mr. Griffiths, and I observed that when he blew his nose, he distinctly passed air through the tympanum. After the consultation, I alluded to his deafness, and the probability, that by a new remedy I could afford him some relief, more especially as he had unconsciously revealed to me, in blowing his nose, a state of ear favourable for success. He readily assented to a trial; and I must be permitted to quote his own statement of the result. On the remedy being applied, he says, "To my utter astonishment I heard every sound so loud, that I felt I had never known what it was to hear until that moment. Sir David Davies could hardly have believed it had he not been present. On entering the streets, the noise was so intense, that I was compelled to stop up my ears to deaden the sound; but after a time I became accustomed to it, and can now enjoy the pleasures of social converse without straining my auricular organs, or being obliged to be addressed in a considerable elevation of voice. Personally I continue to apply the remedy with the same beneficial effect, and am convinced of its permanent nature, when persevered in, and properly attended to. This extraordinary discovery comes too late to be of that essential service it would have been to me in earlier life, yet it may render the rest of my days more comfortable in my intercourse with the world."

The following brief history of Mr. Griffith's case, as detailed by himself, is interesting in many points of view:—The crisis of a severe attack of scarlatina in my infancy was attended by abscesses in both ears, which produced deafness, and a continual discharge of purulent matter, more or less, until I attained my twenty-second year, when the latter ceased. Occasionally concretions of wax formed in the passage, increasing the deafness. These were removed by syringing, after which a thin pellucid fluid would issue from the ears, during which my hearing was much improved, again becoming worse as the discharge ceased. While the discharge lasted I experienced a slight tenderness in my ears, which also ceased with the discharge. I find that your remedy sometimes does the same thing, and that is my reason for not constantly using it; but if it is *not* applied, my hearing is not in the least degree remedied! The discharge is always more profuse when in bed, even without the remedy, and I am somewhat puzzled to account for it. My children know as well as I do when the remedy is applied; and when it is, they remark, 'Your ears are too sharp; we cannot now speak to mamma, even in a whisper; but they cannot, more than other people, discover why I should hear so well one day, and the next, perhaps, not better than usual; and the question now is, 'Have you got your new ears on to-day, papa?' The invention is invaluable."

From this communication, written three or four weeks after his visit to town, it appears that the remedy at first set up an irritation in the ear, which occasionally rendered it advisable that it should be discontinued: but now I am enabled to state that such obstacle to its use no longer exists, and that he applies it regularly, uninterruptedly, and with undiminished success.

This case, like the first quoted, proved to be one in which there was a loss of a great portion of the membrana tympani; and I may here observe, that all my experience tends to show that this is an essential condition of the ear for success. At the present time I can refer to not very far short of two hundred cases, in which the new treatment has been successful, and in all of which more or less perforation or destruction of the membrane exists.

A very small quantity of wool is sufficient. It must be moistened in some fluid without any compression, and gently pushed down the meatus with the point of a probe. I have had constructed for the purpose a set of instruments, which are calculated to meet and overcome every difficulty; for I need scarcely say that it is very easy to talk of passing a foreign body down the meatus, but it is not so easily done. Besides, it is not sufficient to merely pass it down to the site of the membrane; but when there, the spot must be found which it is indispensable the wool should occupy and cover; for then only and not till then, will success attend the application, and the patient regain the hearing.

With a few rules, which, of course, vary with the case, the patient may be taught to manipulate upon himself, and all that is required is to remove the dry wool, and replace it with moist, night and morning, or morning only. This is quite sufficient to maintain the improved hearing in the intervals.

It will be expected that I should say something of the *modus operandi* of this new application; but I can offer nothing that is conclusive. It has appeared to me in some way or other to supply the place of the lost membrane. The moisture is absolutely necessary to its perfect action; for when the wool becomes perfectly dry it impedes rather than improves the power of hearing. Is it possible that moist wool placed at the extremity of the meatus can transmit the vibrations of sound in the same manner as the natural membrane, or must we look for some other explanation? However, of its relieving this kind of deafness there can be no doubt. Some may feel incredulous at such simple means producing such brilliant results; but in order to substantiate that which I have now written, I propose, in my next paper, to quote the statements of my patients, appended to histories of their cases.

The question as to how far this new mode of treatment can be made available in cases in which the membrana tympani remains intact, is now occupying my anxious consideration, and forms the basis of a series of experiments, pending which I will, with the editor's permission, forward to him for publication some observations on "Internal Otorrhœa," and on "Artificial Perforation of the Membrana Tympani."—*Lancet.*

